# Skip lateral lymph node metastasis leaping over the central neck compartment in papillary thyroid carcinoma

**DOI:** 10.18632/oncotarget.15388

**Published:** 2017-02-16

**Authors:** Jianyong Lei, Jinjing Zhong, Ke Jiang, Zhihui Li, Rixiang Gong, Jingqiang Zhu

**Affiliations:** ^1^ Thyroid and Parathyroid Surgery Center, West China Hospital of Sichuan University, Chengdu 610041, China; ^2^ Department of Pathology, West China Hospital of Sichuan University, Chengdu 610041, China

**Keywords:** papillary, thyroid carcinoma, skip metastasis, lymph node, metastasis

## Abstract

**Objective:**

This study was performed to investigate the frequency and pattern as well as the predictive factors of skip metastasis (lateral cervical lymph node metastasis without central lymph node metastasis) in papillary thyroid carcinoma (PTC).

**Methods:**

450 PTC patients who received total thyroidectomy with central neck dissection(CND) combined with modified radical lateral neck dissection(LND) were divided into two groups: with or without skip metastases. The clinicopathological characteristics were statistically compared and analyzed, and univariate and multivariate analyses were performed to detect the risk factors of skip metastasis.

**Results:**

The skip metastasis rate was 8.7% (39/450), and patients with skip metastases had fewer lateral lymph node metastases but were more likely to have single-level lateral metastasis, which are considered Level II(P<0.05). Skip metastasis was significantly associated with the primary tumor location in the upper portion (OR=18.495, 95% CI 6.612-51.731), a primary tumor size ≤10mm (OR=32.492, 95% CI 11.973-88.174) and Capsule invasion (OR=5.822, 95% CI 1.954-17.343) as demonstrated by our prospective study of 10 patients who received an injection of 0.1 ml carbon nanoparticles under ultrasonography in the upper portion of the lobe: 7(70%) had lateral compartment lymph node black staining without ipsilateral center compartment lymph node staining. However, skip metastasis did not affect the PTC patients’ long-term tumor-free survival rate (P=0.432).

**Conclusion:**

Skip metastases can be common, and the primary tumor location in the upper portion, a primary tumor size ≤10 mm, and capsular invasion are closely linked to skip metastasis. The lateral compartment should be carefully evaluated.

## INTRODUCTION

The worldwide incidence of thyroid cancer, especially papillary thyroid carcinoma (PTC), has increased rapidly in recent years [1, 2]. Regional lymph node metastasis is commonly observed in PTC and occurs in approximately 30-80% of PTC patients [3, 4], even in Papillary thyroid microcarcinoma (PTMC, ≤10 mm)[5, 6], which may present an associated increased risk of regional recurrence and distant metastasis [7]. Previous studies reported that nodal metastasis of PTC occurs in a stepwise fashion, with metastasis beginning in the central cervical compartment, continuing to the ipsilateral cervical compartment, and finally arriving at the contralateral lateral or mediastinal compartment; therefore, the lateral compartment is the second site of PTC metastases [8]. However, negative metastasis in the central compartment and positive lateral compartment is often referred to as “skip metastasis”, which is not uncommon in clinical PTC. The frequencies of skip metastasis reported in previous studies ranged from 6.8% to 37.5% [9–11].

To prevent recurrence and metastasis, the preoperative detection of lymph node metastasis and subsequent radical surgical removal must be performed. However, preoperatively detecting lymph node metastases remains a challenge, especially in the central lymph node (CLN) compartment, despite the development of advanced diagnostic tools, such as ultrasonography, enhanced computed tomography (CT) or magnetic resonance imaging (MRI). Skip metastases are more unpredictable [12], and their clinical significance remains unclear. Additionally, few studies have reported the prevalence, characteristics and risk factors of skip metastasis in PTC [9–11]. However, these previous studies were limited by small patient sample sizes and single center analyses. In addition, the skip metastasis rate differed greatly, and the risk factors that were reported varied greatly. Furthermore, credible predictive factors were not reported. In this study, we evaluated the frequency, pattern and predictive factors of skip metastasis within a large cohort of patients and used these data to support our clinical model.

## RESULTS

### Patient demographics

Of the 3668 patients whose medical records were reviewed, 450 met our study criteria and were included in our analysis. The mean age of the patients was 40.5±13.7 years, and the male to female ratio was nearly 1:3 (120:330). The most common race was Han(442 cases, 98.2%). Seventy five patients (16.7%) had chronic diseases, such as hypertension, diabetes, etc. when undergoing surgery; and 8 patients (1.8%) had Graves’ disease; 211 patients(46.9%) had nodular goiters; and 116 patients (25.8%) had autoimmune thyroid disease when PTC was diagnosed (Table 1).

**Table 1:** PTC Patient demographics and clinical characteristics (n=450)

Characteristics	Results
Age at diagnosis (mean±SD, years)	40.5±13.7
≤45 years	295
>45 years	155
Sex (male/female)	120/330
Race (Han/Tibetan/Hui/Yi)	442/6/1/1
Height (cm)	163.7±7.2
Weight (kg)	61.4±10.9
BMI(kg/m^2^)	22.8±3.3
Chronic disease (no/hypertension/diabetes/both/other)	375/60/6/4/5
Autoimmune thyroid disease (yes/no)	116/334
Graves’ disease (yes/no)	8/442
Nodular goiters (yes/no)	211/239
NLR(mean±SD)	1.9±0.9
≤2	291
>2	159
PLR(mean±SD)	108.1±50.5
≤200	218
>200	232
TSH level(mU/L, mean±SD)	3.2±3.0
FT4 level(pmol/L, mean±SD)	17.5±6.1
FT3 level(pmol/L, mean±SD)	4.9±0.7

Abbreviations: SD: standard deviation; BMI: body mass index; NLR: neutrophil-to-lymphocyte ratio; PLR: platelet-to-lymphocyte ratio; TSH: thyroid stimulating hormone; FT3: free triiodothyronine; FT4: free thyroxine

### Tumor characteristics

As shown in Table 2, 74 (16.4%) and 71 patients (15.8%) presented multifocality and bilaterality, respectively. Papillary thyroid microcarcinoma (PTMC) was found in 96 patients(21.3%). Pathological capsular invasion and extrathyroid extension was detected in 250 (55.6%) and 130 (28.9%) patients, respectively. PTCs were nearly evenly distributed in the three equal lobe areas, with 178(39.6%) in the upper portion, 133 (29.6%) in the middle portion, 121(26.9%) in the lower portion, and 18(4%) were observed in the isthmus. Most of PTC cases were T3 (282 cases, 62.7%), and 92 patients (20.4%) had T4 because of the invasion of the recurrent laryngeal nerve (RLN), trachea, esophagus or prevertebral fascia. Additionally, 12 cases (3.3%) were preoperatively diagnosed with distant metastasis, including 9 cases in the lung, 1 case in bone, and 2 cases in both lung and bone.

**Table 2:** PTC tumor characteristics and lymph node metastasis(n=450)

Characteristics	Results
Multifocality (yes/no)	74/376
Bilaterality (yes/no/isthmus/isthmus+lobe)	71/359/8/12
Capsule invasion (yes/no)	250/200
Extrathyroid extension (yes/no)	121/329
Total tumor size (mean±SD, cm)	17.1±9.7
Total tumor size (≤10 mm, >10 mm)	96/244
Largest tumor size (mean±SD, mm)	16.2±10.4
Primary tumor location	
Upper	178
Middle	133
Lower	121
Isthmus	18
Tumor extension	
T1	58
T2	18
T3	282
T4	92
Preoperative distant metastasis (no/lung/bone/both)	438/9/1/2
Central neck node number	
Harvested	11.0±3.0
Metastatic	5.9±3.2
Ipsilateral central neck node number	
Harvested	6.3±2.1
Metastatic	3.5±2.1
Lateral neck node	
Harvested	25.9±11.2
Metastatic	6.4±2.8
Lateral neck node metastasis cases	
Level II	184(40.9%)
Level III	339(75.3%)
Level IV	328(72.9%)
Level V	173(38.4%)

### Pattern of lymph node metastasis

The mean number of total central neck lymph nodes was 11.0±3.0, of which 5.9±3.2 had metastases. Of these nodes, the mean number of ipsilateral central neck nodes was 6.3±2.1, of which 3.5±2.1 had metastases. Skip metastases were found in 39 patients (8.7%). In the lateral compartment, the mean number of harvested lymph nodes was 15.9±7.2, of which 6.4±2.8 had metastases, and the most frequently involved sites were Levels III(75.3%) and IV(72.9%) followed by Levels II and V (as shown in Table 2). A further analysis revealed that double-level metastasis (181 cases, 40.2%) was the most common model for lateral compartment metastasis, followed by triple-level metastasis (129 cases, 28.7%), single-level metastasis (95 cases, 21.1%) and four-level metastasis (45 cases, 10%) (Table 3).

**Table 3:** Comparison of clinicopathological variables between two groups of patients with PTC

Variable	Skip Metastasis		P-value
	Present (n=39)	Absent (n=411)	
Age (≤45/>45 years)	29/10	266/145	0.227
Sex (male/female)	12/27	108/303	0.545
Race (Han/Other)	38/1	404/7	0.698
BMI (<24/≥24 kg/m^2^)	29/10	272/139	0.300
Chronic disease(yes/no)	10/29	65/346	0.116
Autoimmune thyroid disease (yes/no)	10/29	106/305	0.984
Graves’ disease (yes/no)	3/36	5/406	0.055
Nodular goiters (yes/no)	24/15	257/154	0.903
NLR (≤2/>2)	29/10	263/148	0.195
PLR (≤200/>200)	39/0	391/20	0.159
TSH level (≤4.2/>4.2 mU/L)	31/8	332/79	0.845
Multifocality (yes/no)	38/1	338/73	0.015
Bilaterality (yes/no)	36/3	383/28	0.596
Capsule invasion (yes/no)	33/6	217/194	<0.001*
Extrathyroid extension (yes/no)	12/27	109/302	0.615
Total tumor size (≤10 mm, >10 mm)	28/11	67/344	<0.001*
Primary tumor size (≤10 mm, >10 mm)	28/11	92/319	<0.001*
Tumor extension (T1-T2/T3-T4)	6/33	76/335	0.631
Primary Tumor	37/2/0/0	141/131/121/18	<0.001*
location(Upper/Middle/Lower/Isthmus)			
Preoperative distant metastasis (yes/no)	0/39	12/399	0.280
Central neck node number			
Harvested	10.8±2.9	11.0±3.0	0.693
Metastatic	0.3±0.6	6.4±2.8	<0.001
Ipsilateral central neck node number			
Harvested	6.8±1.9	6.3±1.7	0.095
Metastatic	-	3.8±1.9	<0.001
Lateral neck node metastasis LN number	5.4±2.2	6.5±2.9	0.015
Lateral neck node metastasis LN cases			
Level II	26(66.7%)	158(38.4%)	<0.001
Level III	23(59.0%)	316(87.6%)	0.041
Level IV	2(5.1%)	326(79.3%)	<0.001
Level V	1(2.6%)	177(43.1%)	<0.001
Lateral compartment single level metastasis	27(69.2%)	68(16.5%)	<0.001
Lateral compartment double levels metastasis	11(28.2%)	170(41.4%)	0.110
Lateral compartment triple levels metastasis	1(2.6%)	128(31.1%)	<0.001
Lateral compartment four levels metastasis	0	45(10.9%)	0.030

Abbreviations: BMI: body mass index; NLR: neutrophil-to-lymphocyte ratio; PLR: platelet-to-lymphocyte ratio; TSH: thyroid stimulating hormone;

### Clinicopathological factors of skip metastasis in the lateral neck

Significant clinicopathological factors that affect skip metastases occurring in the lateral compartment without central neck metastasis were identified and compared between the two groups in Table 3. The PTCs with skip metastases were more commonly detected with capsular invasion (84.6% vs 52.8%, P<0.001). A total tumor size≤10 mm was more common in the skip metastases group (71.8% vs 16.3%, P<0.001), and a similar trend was observed for primary tumor size ≤10 mm (71.8% vs 22.4%, P<0.001). The skip metastases group showed more cases with a primary tumor located in the upper portion (94.9% vs 34.3%, P<0.001), and almost all of the PTC skip metastases were located in the upper portion except for 2 cases located in the middle portion (as shown in Table 3).

The lymph node metastasis rate and characteristics are shown and compared in Table 3. The PTC skip metastases showed a lower central neck node metastasis number (P<0.001) and ipsilateral central neck node metastasis number(P<0.001), even with comparable numbers of harvested nodes. Seven patients presented contralateral central lymph node metastasis with 10 lymph nodes overall, even without ipsilateral central neck lymph node metastases. In addition, the PTC skip metastasis presented a much lower lateral neck node metastasis LN number (5.4±2.2 vs 6.5±2.9, P=0.015). The PTC skip metastases showed much higher Level II metastasis but much lower Level III, IV, and V metastases (all P<0.05). The PTC skip metastases showed a much higher number of single-level metastases in the lateral compartment, much lower triple- and quadruple-level metastases in the lateral compartment (P<0.05) and comparable amounts of double-level metastases in the lateral compartment(P=0.110).

### Predictive factors of skip metastases

**Figure 1:**
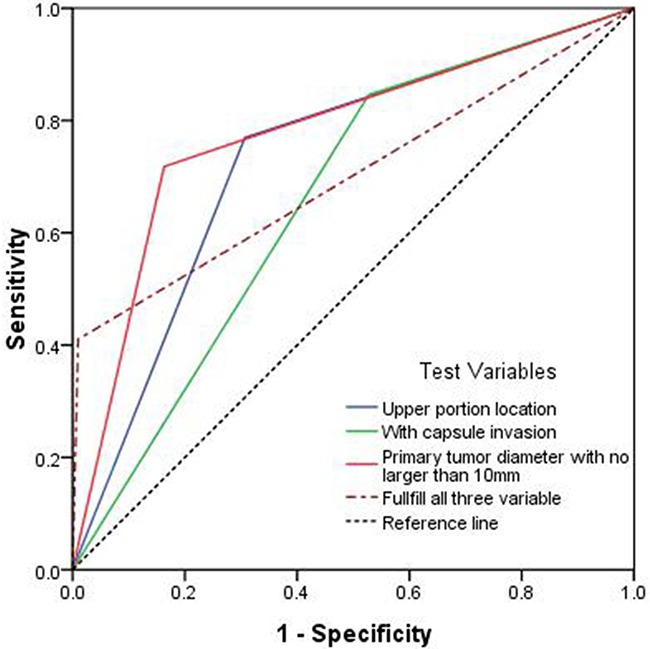
Predictability of skip metastasis shown by a receiver operating characteristic (ROC) curve for primary tumors with a diameter no larger than 10 mm (AUC: 0.777), tumors located in the upper portion location (AUC: 0.731) and tumors presenting capsular invasion (AUC: 0.659)

The associations between skip metastases and the primary tumor location and size and capsular invasion were statistically significantly using univariate and multiple logistic regression analyses as shown in Table 4. The ORs for the primary tumor location in the upper portion, primary tumor size ≤10 mm and capsular invasion were 18.495 (95% CI 6.612-51.731), 32.492 (95% CI 11.973-88.174) and 5.822 (95% CI 1.954-17.343), respectively. A receiver operating characteristic (ROC) curve analysis was performed to determine the predictability of skip metastasis according to the primary tumor location in the upper portion, primary tumor size ≤10 mm, and capsular invasion and ascertain whether the cases fulfilled all three variables. Skip metastases were best predicted according to a primary tumor diameter no larger than 10 mm (AUC: 0.777) and then according to an upper portion location (AUC:0.731) and capsular invasion (AUC: 0.659). When the cases fulfilled all three risk factors, the specificity was 0.98, and the AUC was 0.700, as shown in Figure 1.

**Table 4:** Multivariate analyses of factors contributing to skip metastasis of PTC

Variables	Odds ratio	95% CI	P-value
Capsule invasion (yes/no)	5.822	1.954-17.343	0.002*
Primary tumor size (≤10 mm/>10 mm)	32.492	11.973-88.174	0.006*
Total tumor size (≤10 mm/>10 mm)	4.686	0.143-153.101	0.385
Primary tumor location(upper/other sites)	18.495	6.612-51.731	<0.001*

**Figure 2:**
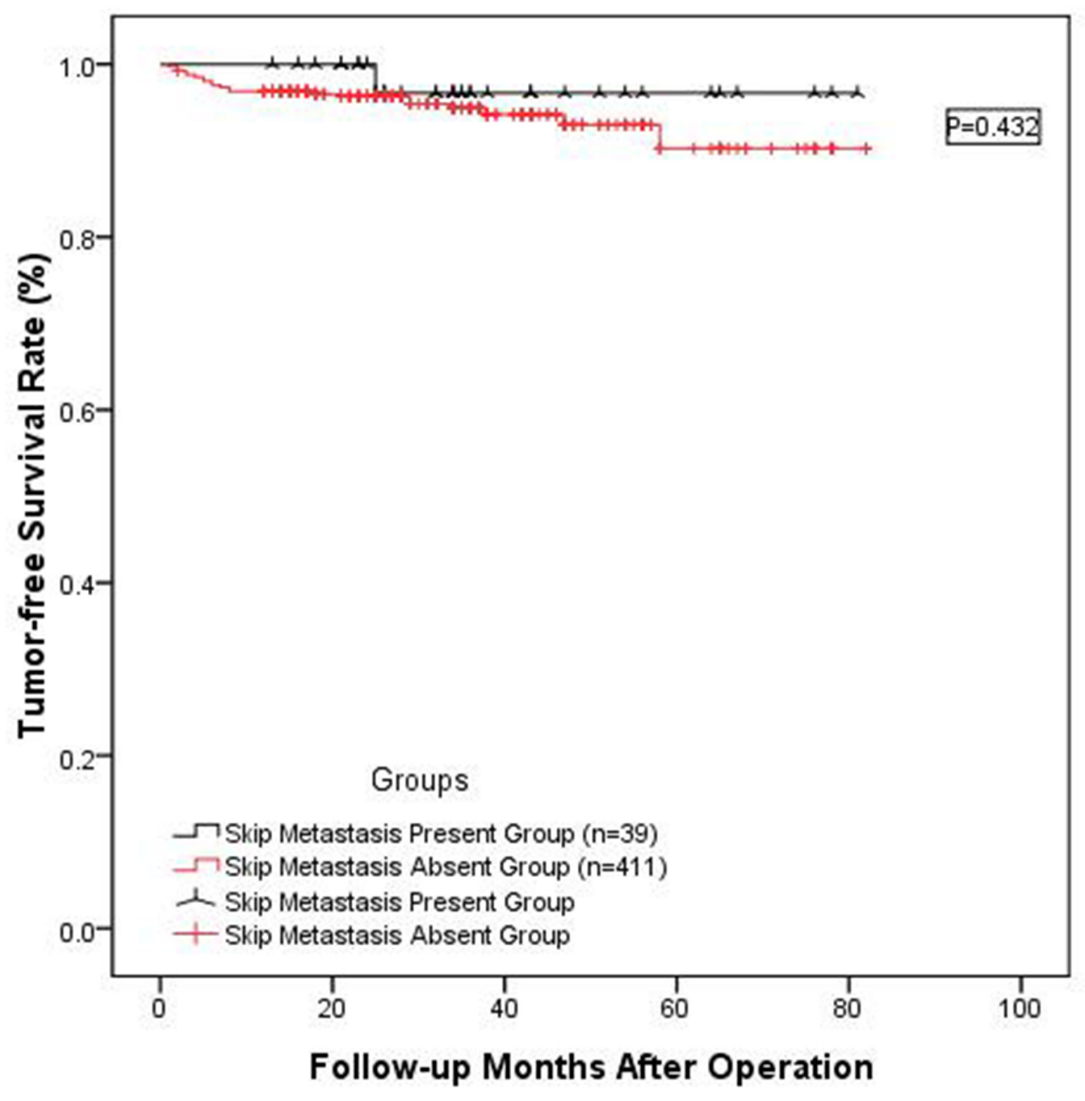
Long-term tumor-free survival rate between 39 patients presenting skip metastasis and the 411 patients without skip metastasis The two groups of patients did not show significant differences (P=0.432).

In the 39 patients with skip metastasis, one patient suffered postoperative hypoparathyroidism and recovered 8 months later and one patient suffered from wound infection. In addition, we compared the tumor-free survival of the two patient groups. As shown in Figure 2, significant differences were not observed (P=0.432); therefore, skip metastasis should not lead to tumor recurrence or affect the patient's long term survival.

### Clinical model of skip metastasis

Ten PTCs were injected with 0.1 ml carbon nanoparticles into the upper portion of the lobe, and the black-stained lymph nodes were counted and are shown in Table 5. Overall, 7 (70%) patients did not show black staining of the central lymph node. However, for the lateral lymph nodes (skip black staining), all 10 patients (100%) presented black staining in the Level II metastases, 9 patients (90%) presented staining in the Level III and IV metastases, and 7 patients (70%) presented staining in the Level V metastases. In addition, Level II (61.7%) had the highest lymph node black staining rate followed by Levels III (55.7%), V(42.9%) and IV(33.3%).

**Table 5:** Prospective study on skip metastasis using carbon nanoparticles

patients	Total harvest lymph node number	ipsilateral central harvest lymph node number	ipsilateral central lymph node black staining number(%)	ipsilateral lateral harvest lymph node number	ipsilateral lateral lymph node black staining number(%)	Level II (%)	Level III(%)	Level IV(%)	Level V(%)	Skip black staining (yes/no)
1	62	8	1(12.5%)	54	25(46.3%)	5(50%)	0(0%)	0(0%)	20(80%)	No
2	24	3	0(0%)	21	10(47.6%)	4(57.1%)	3(75%)	1(14.3%)	2(66.7%)	Yes
3	28	7	0(0%)	21	8(38.1%)	2(66.7%)	4(80.0%)	1(16.7%)	1(14.3%)	Yes
4	21	5	3(60%)	16	12(75%)	2(100%)	3(75%)	5(83.3%)	2(50%)	No
5	21	4	1(25%)	17	7(41.2%)	1(33.3%)	4(100%)	2(40%)	0(0%)	No
6	27	6	0(0%)	21	11(52.4%)	2(66.7%)	5(100%)	4(50%)	0(0%)	Yes
7	29	5	0(0%)	24	12(50%)	4(100%)	5(83.3%)	3(37.5%)	0(0%)	Yes
8	39	8	0(0%)	31	11(35.5%)	3(60%)	5(55.6%)	2(16.7%)	1(20%)	Yes
9	29	5	0(0%)	24	14(58.3%)	4(80%)	4(50%)	4(66.7%)	2(40%)	Yes
10	30	3	0(0%)	27	7(25.9%)	2(40%)	1(16.7%)	2(25%)	2(25%)	Yes
Average	31	5.4	0.5(9.3%)	25.6	11.7(45.7%)	2.9(61.7%)	3.4(55.7%)	2.4(33.3%)	3(42.9%)	7(70%)

## DISCUSSION

Lymph node metastasis is common for PTCs and accounts for 30%-80% of all PTCs, including PTMC [13]. Surgeons frequently elect to perform radical surgery of the metastasized lymph; however, reoperation for PTC recurrence may significantly increase the surgical complications and medical costs and affect the patient's quality of life [14, 15]. Therefore, preoperative evaluations of lymph node metastasis must be performed to determine the exact extent of neck dissection. The thyroid gland has been reported to contain its own internal lymphatic system and external lymphatic system. Central lymph nodes are the most frequent nodes involved inmetastasis followed by the lateral lymph nodes, which indicates that cervical lymph node metastasis occurs in a stepwise fashion. Lymphatic drainage of the thyroid occurs first in the central compartment (Level VI) and then in the lateral neck compartment.

Compared with the general patterns of thyroid lymphatic drainage, skip metastasis, which is defined as negative ipsilateral central and positive ipsilateral lateral compartment lymph nodes, is a specific but not uncommon type of metastasis in patients with PTC [9–11, 16–21] and MTC [20, 22]. The significance of skip metastasis in PTC patients is still unknown. Compared with the results of previous studies [9–11, 16–21], the present study first evaluated and compared the impact of skip metastases on tumor recurrence and survival and obtained negative results that were inconsistent with the results of previous studies on non-small-cell lung cancer and colorectal cancer, which indicated that skip metastasis may have a positive impact on prognosis [23, 24]. The negative impact of skip metastasis on recurrence and survival reported here was likely related to the limited incidence of lymph node metastases as shown in Table 3.

The incidence of skip metastasis in PTC patients ranges from 3.0% to 19.7% [9–11, 16–21]. However, these results were limited by the small sample size of the studies, which had a maximal patients number of 147. This number is only one third the size of our sample (Table 6). In addition, almost all of the studies were performed in Korea except for two studies. This study is the first report from the Chinese mainland, which accounts for almost 20% of PTC cases worldwide [1]. The rate of skip metastasis in our present study was 8.6% (39/450), which is comparable to that of certain studies [11, 18, 19, 21]] but much lower than other reports [9, 10, 16, 17, 20]. The possible reason for this difference could be heterogeneous patient populations in the samples [9]. In addition, our study first reported 7 cases with contralateral central lymph node metastasis (10 lymph nodes metastases overall) and ipsilateral lateral neck lymph node metastasis but without ipsilateral central neck lymph node metastasis, which may indicate migration via the lymphatic drainage across the center median to the contralateral central part (VI region).

**Table 6:** Literature review for skip metastasis in PTCs

Author	Country	Publication year	Included patients	Skip rate(%)	Risk factors
Machens et al [20]	Germany	2004	66	13(19.7%)	No features
Chung et al [21]	Korea	2009	39	3(7.7%)	No features
Koo et al [16]	Korea	2010	45	12(17.1%)	Multifocal primary tumor; positive LN involvement in all lateral levels
Lee et al [19]	Korea	2007	46	1(3.0%)	No description
Roh et al [18]	Korea	2008	52	5(9.6%)	No description
Lim et al [9]	Korea	2012	90	17(19.0%)	Fewer lymphovascular invasion; extracapsular spread
Kliseska et al [17]	Croatia	2012	42	8(19.5%)	No description
Park et al [10]	Korea	2012	147	32(21.8%)	Upper pole; tumors≤10 mm;Unifocal
Lee et al [11]	Korea	2013	131	9(6.9%)	Upper part
Present study	China	2016	450	39(8.7%)	Capsule invasion; Primary tumor size≤10 mm; upper portion location

The risk factors associated with skip metastasis have been discussed in previous studies (Table 6). However, these factors were not observed in two studies [20, 21]; therefore, these studies did not include risk factors related to skip metastasis. Similar to previous studies [10, 11], we found that a primary tumor located in the upper portion was a predictive factor. This predictive factor could be explained by the nature of the lymphatic drainage system of the thyroid gland because tumor cells from PTC located in the upper lobe were more likely to be transported to the lateral lymph nodes along the superior thyroid artery [25] and tumor cells from the mid-lower region of the gland were more likely to be transported to the CLN. The lymphatic drainage system explains why skip metastasis frequently occurred in the upper lobe PTCs(39 cases) except for two cases in which the tumors were located in the middle portion. In addition, lateral node metastasis preferentially occurred along the lymphatic chain as reported by previous studies. Level III nodes were the most frequently involved nodes, followed by Levels IV, II and V [18]. However, the 39 patients with lymph node skip metastasis had a much higher rate of Level II but a lower rate of Level IV and V lymph node involvement, which was mainly because of location of the primary tumors on the upper portion of the gland. In addition, our results indicated that the 39 cases with skip metastasis showed a greater number of single-level metastases and fewer triple- and quadruple-level metastases, which may be related to the smaller tumor size and upper portion tumor location. In addition to the upper portion, a primary tumor size no larger than 10 mm was another risk factor because skip metastasis is more frequent in less aggressive PTCs, such as PTMC [9, 20]. In addition, our study showed that skip metastasis was much more common in PTC patients with primary tumor capsular invasion, which was consistent with a previous study [9]. Lymphatic systems that bypass the central lymph node compartment may represent the most likely hypothesis for skip metastasis. However, false negative findings caused by limited lymph node sampling or misdiagnoses during routine histopathology may also contribute to the incidence of skip metastasis [9, 26].

All three risk factors related to skip metastasis were observed using our simulation experiments in which 0.1 ml of carbon nanoparticles were injected into the upper portion of the lobe in ten patients. The rate of black staining in the central and lateral lymph nodes was calculated, and the results showed that 70% of the patients showed skip black staining, all of the patients showed Level II lymph node black staining, and 90% of patients had Level III and IV, and 70% of patients had Level V lymph node black staining, respectively. The results of our prospective simulation experiment were consistent with the results of a retrospective study and further supported our conclusions. Carbon nanoparticles have been used to guide central neck dissection [27] or identify and protect the parathyroid [28]. However, this study is the first to use this method to simulate skip metastasis and confirm the risk factors. The diameter of the carbon nanoparticles is 150 nm [28], which is much smaller than the diameter of a tumor cell. Therefore, lymph node black staining is able to simulate the metastasis of tumor cells to lymph nodes. However, additional investigations are needed to confirm our results.

Our study was limited by its retrospective nature. In addition, our study enrolled only PTC patients who accepted therapeutic lateral compartment dissection (with FNAC-proven metastasis), which is similar to previous reports. FNAC had nearly 10-20% false negatives, and latent lateral metastases were detected in more than 50% of the patients who received prophylactic lateral compartment dissection [29]. Additional factors and more accurate detection are required to predict skip metastasis after prophylactic lateral compartment dissection, which is not recommended by ATA, ETA or CTA guidelines.

In conclusion, the rate of skip metastasis in the patients presenting PTC was 8.7%. Therefore, the lateral compartment should be carefully examined for skip metastases, especially for PTC patients who present primary tumors in the upper portion, have a primary tumor size ≤10 mm or show invasion of the tumor capsule.

## MATERIALS AND METHODS

This study was approved by the local institutional review boards, and approval and informed consent were obtained from all patients before the study began. The postoperative histology medical records of all of the PTC patients who underwent simultaneous total thyroidectomy with the central compartment LND and ipsilateral therapeutic lateral compartment LND were reviewed and analyzed. Cases with one of the following items were excluded from our study: revision surgery, history or presence of other head and neck carcinomas, other types of thyroid carcinoma, negative lateral lymph node metastases in postoperative histopathologic examination, focal “berry-picking” for CLN or LLN compartments, or less than total thyroidectomy combined with bi-CLN and ipsilateral LLN dissection.

Preoperative ultrasonography and neck enhanced CT and/or MRI were performed by experienced technicians and used to evaluate the tumor size, location, presence of nodal metastases and other tumor characteristics. The imaging results were co-evaluated by two readers: a radiologist with at least 15 years of experience and a surgeon with at least 20 years of experience. For the suspicious PTCs (i.e., central necrosis or cystic change, dense cortical enhancement, or calcification), fine-needle aspiration biopsy (FNAB) and cytological BRAF^V600E^ mutation were performed to confirm the diagnosis. All lateral neck lymph node compartment dissections were performed for PTC patients who were confirmed to have clinical evidence of positive lateral neck nodes (no prophylactic lateral lymph node dissection) via FNAB or thyroglobulin assessment. The histopathological evaluation of the thyroid specimens was performed by experienced pathologists who had at least 10 years of experience and had diagnosed over 500 PTC cases.

The number of LNMs was calculated and analyzed with respect to neck level. The lateral compartment and central compartment were divided into four sub-parts: ipsilateral and contralateral central compartments as well as prelaryngeal (Delphian) and pretracheal compartments. Superior central lymph node compartment dissection was performed laterally on the thyroid cartilage notch to the carotid sheaths, posteriorly to the prevertebral fascia, including VI A (superficial right recurrent laryngeal nerve) and VI B (posterior of right recurrent laryngeal nerve) and inferiorly to the innominate vein. The lateral cervical lymph nodes were classified into four neck levels (II-V) based on the criteria from the American Head and Neck Society [30] and American Thyroid Association (ATA)[31]. In every case, the lateral compartment was radically modified superiorly to the posterior belly of the digastric muscle, inferiorly to the subclavian vein, and laterally to the anterior border of the trapezius muscle. The central and lateral compartments were removed in whole pieces and then divided into sublevels according to the intraoperative mark.

CLN dissection was performed after the total thyroid was removed, bilateral CLN dissection was performed routinely, and radical lymph node dissection, including the lateral lymph node compartment, was performed for radical therapeutic purposes [32]. Tumor locations were categorized as the isthmus and theupper, middle, and lower portion based on the results of the preoperative ultrasonography with a longitudinal view. In multifocal cases, the analysis was based on the largest tumor diameter. In our study, skip metastasis was defined according to previous reports [9–11] as an ipsilateral lateral lymph node metastasis with no positive nodes in the central compartment.

Clinical and pathological variables that may be related to skip metastasis were compared between the skip metastasis group and the control group. All variables with a value of P<0.05 were included in the multivariate analysis, and comparisons were performed among the predictive factors using the ROC Curve. To verify our predictive factors, we performed prospective research on 10 PTC patients who presented with preoperative FNAC-supported lateral metastasis and PTCs located in middle or lower portion of the lobe. When freeing flaps is ready, ultrasonography was used to guide an injection of 0.1 ml carbon nanoparticles into the upper portion of the lobe, with nearly 0.5cm maintained between the injection site and the thyroid capsule (anterior, lateral and posterior)[28]. The diffusion diameter of the 0.1 ml carbon nanoparticles in the thyroid gland under ultrasonography was approximately 10 mm. The surgical procedure was performed routinely 10 minutes after the injection, and the total number of harvested and black-stained lymph nodes of the ipsilateral center compartment and sub-levels of the lateral compartment were separately counted by two surgeons and then by one experienced pathologist. If one lymph node was not stained completely, it was counted as a stained lymph node. When the number of lymph nodes was inconsistent, the investigators were either consulted or the average value of all evaluations was used.

Continuous and categorical data were expressed as the mean±SD and the rates, respectively. The differences between continuous data and categorical data were compared and analyzed using the Mann-Whitney U test and the chi-square or Fisher's exact test (2-tailed) if necessary. Univariate analyses were performed for the association between skip metastases and clinicopathological factors using the Pearson chi-square test or Fisher's exact test. All of the variables with values of P<0.05 were included in the multivariate analysis to assess the independent predictive factors using a Cox regression analysis. The odds ratios (ORs) and 95% relative confidence intervals (CIs) were calculated to determine the relevance of all potential predictors. The predictability of skip metastasis according to the multivariate analysis was assessed using the ROC curve. A 2-tailed P-value of <0.05 was considered significant. SPSS software (SPSS 17, Inc., Chicago, IL, USA) was used to manage the data and for the statistical analyses.

## Abbreviations

PTC: papillary thyroid carcinoma
CND: central neck dissection
LND: lateral neck dissection
CT: computed tomography
MRI: magnetic resonance imaging
ATA: American Thyroid Association
FNAB: fine-needle aspiration biopsy
PTMC: Papillary thyroid microcarcinoma
